# Molecular Characterization of Polyomavirus-Positive and Negative Merkel Cell Carcinoma

**DOI:** 10.3390/cancers17213508

**Published:** 2025-10-31

**Authors:** Poorva Vaidya, Sharon Wu, Dave Bryant, Curtis J. Perry, Varsha Prakash, Emil Lou, Theresa Guo, Isaac Brownell, Sourat Darabi, Ling Gao, Farah Abdulla, Soo J. Park

**Affiliations:** 1 Division of Hematology/Oncology, UC Irvine School of Medicine, Orange, CA 92868, USA; poorvav@hs.uci.edu; 2 Caris Life Sciences, Phoenix, AZ 85001, USA; swu@carisls.com (S.W.); dbryant@carisls.com (D.B.); fabdulla@carisls.com (F.A.); 3 Division of Medical Oncology and Hematology, Yale School of Medicine, New Haven, CT 06520, USA; curtis.perry@yale.edu; 4 Oregon Health & Science University, Portland, OR 97239, USA; prakashv@ohsu.edu; 5 Division of Hematology/Oncology and Transplantation, Department of Medicine, Medical School, University of Minnesota, Minneapolis, MN 55455, USA; emil-lou@umn.edu; 6 Department of Otolaryngology-Head & Neck Surgery, University of California, San Diego, CA 92093, USA; twguo@health.ucsd.edu; 7 Dermatology Branch, National Institute of Arthritis and Musculoskeletal and Skin Diseases, Bethesda, MD 20891, USA; isaac.brownell@nih.gov; 8 Hoag Family Cancer Institute, Newport Beach, CA 92663, USA; sourat.darabi@hoag.org; 9 Department of Dermatology, UC Irvine School of Medicine, Orange, CA 92697, USA; lingg3@hs.uci.edu; 10 Division of Hematology-Oncology, UC San Diego School of Medicine, La Jolla, CA 92093, USA

**Keywords:** Merkel cell carcinoma, immune checkpoint inhibitors, Merkel cell polyomavirus, oncology, drug response, whole transcriptome sequencing

## Abstract

**Simple Summary:**

Unresectable and metastatic Merkel Cell Carcinoma is treated with immune checkpoint inhibitors, regardless of Merkel Cell polyomavirus status. However, many cases are either refractory to checkpoint inhibition or develop resistance after initial response. There remains an unmet clinical need to identify alternative therapeutic targets. Prior studies have implicated distinct molecular profiles of virus-positive and virus-negative disease. Here, whole exome sequencing confirmed known mutation differences between virus-positive and virus-negative disease, while whole transcriptome sequencing identified key differences in MAPK Pathway Activity Score (MPAS), NK cell infiltration, and expression of immune checkpoint genes.

**Abstract:**

Background/Objectives: Immune checkpoint inhibitors (ICIs) are frontline treatment for advanced Merkel Cell Carcinoma (MCC), regardless of viral status. Frontline ICIs provide durable benefit to only half of patients, highlighting a need for alternative therapies. In this study, the objective is to leverage whole exome sequencing (WES) and transcriptome sequencing (WTS) to distinguish genomic alterations associated with ICI response. Investigate differential genomic alterations between virus-positive (VP) and virus-negative (VN)-MCC to identify novel therapeutic targets. Methods: A total of 95 MCC cases underwent WES and WTS. Utilizing computational pipelines applied to WES, we identified viral status and tumor mutational burden (TMB). RNA-seq data was used to characterize the immune microenvironment. Results: Of 95 MCC cases, 57 (60%) were VP-MCC and 38 (40%) were VN-MCC. Median TMB was higher in VN-MCC (27.5 vs. 1 Muts/Mb). Mutations in *TP53*, *RB1*, *NOTCH1*, *KMTD2*, *KMT2C*, and *PIK3CA* were primarily found in VN-MCC. MAPK Pathway Activity Score, NK cell infiltration, and the immune checkpoint gene *CD276* in VN-MCC tumors were upregulated. No overall survival (OS) difference was identified between VP and VN-MCC, even after ICIs. Conclusions: MCC oncogenesis and treatment response transcend viral status. While mutational analysis confirms previous findings, assessment of the transcriptome and tumor microenvironment suggests alternate therapeutic targets.

## 1. Introduction

Slated to affect 3284 patients per year in the United States by 2025, Merkel Cell Carcinoma (MCC) is a rare, aggressive skin cancer [1,2]. In 2008, it was demonstrated that the Merkel Cell Polyomavirus (MCPyV) plays a key role in MCC oncogenesis and is found in 80% of tumors, while ultraviolet radiation (UVR) plays a role in the oncogenesis of virus-negative (VN) tumors [3]. Additional risk factors for the development of MCC include immunosuppression (e.g., solid-organ transplant, hematologic malignancy, or HIV/AIDS) and advanced age [2,4]. Historically, advanced MCC was treated with chemotherapy, but outcomes were poor, with progression-free survival (PFS) of 3 months or less [5]. Clinical outcomes have significantly improved as treatment paradigms have shifted toward frontline immune checkpoint inhibitors (ICIs) for both virus-positive (VP) and VN tumors, with five-year overall survival doubling across metastatic disease [6]. However, a significant portion of VP and VN-MCC patients have disease refractoriness or develop resistance to ICIs [7,8,9,10]. Additionally, some patients may have contraindications or intolerance to ICIs [11,12]. Thus, there remains a need to further distinguish molecular features of MCC that predict response to treatment or identify alternate therapeutic targets.

Prior studies have leveraged methodologies ranging from next-generation sequencing (NGS) to immunofluorescence to identify differences between ICI-responsive and ICI-refractory disease [13,14]. Multiplex immunofluorescence staining demonstrated a predominance of CD8+ effector and central memory T-cells in close proximity to tumor cells, which was associated with a favorable response [14]. Single-cell RNA sequencing (scRNA-seq) with spatial transcriptomics demonstrated that responders have increased type I/II interferons and pre-existing tissue-resident CD8 or Vδ1 γδ T cells [13]. RNA sequencing demonstrated genes pertaining to angiogenesis and MAPK pathways are upregulated in cases with poor survival, while genes pertaining to immune response are upregulated in survivors [15]. DNA methylation signatures in MCC tumorigenesis have implicated epigenetic factors [16]. NGS has also been leveraged to identify viral integration sites in VP-MCC to distinguish between viral oncogenesis and components of UV-driven oncogenesis [17]. While many of these studies have provided insight into pathogenesis and alternate biomarkers, no robust predictive biomarker or alternate therapeutic target has been clearly established in treatment paradigms [18,19]. Here, we further the effort to molecularly characterize MCC with whole exome sequencing (WES), whole transcriptome sequencing (WTS), and comprehensive tumor microenvironment analysis, which has not been previously reported in MCC and may provide insight into treatment response and potential drug targets. Using WES and WTS, we aimed to 1) define and quantify immune pathway activation and 2) determine differential gene pathway expression in both VP and VN-MCC. We further investigated gene alteration patterns associated with prognosis and treatment response to systemic regimens with the aim of identifying potential predictive biomarkers.

## 2. Materials and Methods

### 2.1. Sample Collection from Participants

A total of 95 Merkel Cell Carcinoma (MCC) tumors underwent comprehensive tumor profiling at Caris Life Sciences (Phoenix, AZ, USA). Pathological diagnosis of MCC was initially determined by the submitting institution, with secondary independent validation conducted by pathologists at Caris Life Sciences. This study was conducted in accordance with the guidelines of the Declaration of Helsinki, Belmont Report, and U.S. Common Rule. In keeping with 45 CFR 46.104 (d), this study was performed utilizing retrospective, deidentified clinical data from patients with MCC. Analysts did not have access to de-identified data, including the institution of origin. Therefore, this study was deemed Institutional Review Board exempt, and no patient consent was necessary from the subjects.

### 2.2. DNA Sequencing

Whole exome sequencing (WES) was performed on genomic DNA isolated from formalin-fixed paraffin-embedded (FFPE) tumor samples. All variants were detected with >99% confidence based on allele frequency and amplicon coverage, with an average sequencing depth of coverage of >500 and an analytic sensitivity of 5%. Whole transcriptome sequencing was performed using RNA isolated from FFPE samples. More than 700 clinically relevant genes at high coverage and high read depth were used, along with another panel designed to enrich for an additional >20,000 genes at lower depth. All variants were detected with >99% confidence based on allele frequency and amplicon coverage, with an average sequencing depth of coverage of >500 and an analytic sensitivity of 5%. Prior to molecular testing, tumor enrichment was achieved by harvesting targeted tissue using manual microdissection techniques. Genetic variants were identified according to the American College of Medical Genetics and Genomics (ACMG) standards, with ’pathogenic,’ and ‘likely pathogenic’ genes counted as mutations.

### 2.3. Global Loss of Heterozygosity (gLoH)

The 22 autosomal chromosomes were split into 552 segments (2–6 Mb in size), and the LoH of single-nucleotide polymorphisms (SNPs) within each segment was calculated. Caris WES data consist of approximately 250 K SNPs spread across the genome (17 SNPs/Mb), with 200 K from exonic regions and 50 K from intronic regions (Agilent Technologies, Santa Clara, CA, USA). The MI Tumor Seek Hybrid assay uses a KAPA HyperChoice MAX 5 Mb Baits SNP panel (Roche, Indianapolis, IN, USA). SNP distances of each region were compared to control distances of a non-LoH reference (NA12878; RRID:CVCL_7526) via Student’s *t*-test. A region was called positive if the average distance was larger than 0.15 Mb and the corrected *p*-value was less than 0.02. Segments excluded from the calculation of genomic LoH include those spanning ≥ 90% of a whole chromosome or chromosome arm and segments that are not covered by the SNP backbone and the WES panel. The final call of genomic LoH is based on the percentage of all 552 segments with observed LoH (high ≥ 16%, low < 16%). For MI Tumor Seek Hybrid only, an indeterminate result is reported if fewer than 3000 SNPs can be read or sample depth is <200×.

### 2.4. COSMIC Mutational Signatures

Single-base substitutions detected by whole exome sequencing were used to evaluate similarity between the patient’s mutation profile and established molecular phenotypes [20].

### 2.5. Merkel Cell Polyomavirus Detection

A total of 91 cases submitted to Caris Life Sciences as MCC were stained with MCPyV IHC (clone CM2B4) and used as the orthogonal testing method for comparison to the MI Tumor Seek-Hybrid Pathogen Panel that included DNA sequencing baits for the detection of MCPyV. The overall percent agreement between the two assays was 94.3%.

### 2.6. RNA Sequencing

FFPE specimens underwent pathology review to measure percent tumor content and tumor size; a minimum of 10% of tumor content in the area for microdissection was required to enable enrichment and extraction of tumor-specific RNA. The Qiagen RNA FFPE tissue extraction kit (Qiagen, Hilden, Germany) was used for extraction, and the RNA quality and quantity were determined using the Agilent TapeStation (New York, NY, USA). Biotinylated RNA baits were hybridized to the synthesized and purified cDNA targets, and the bait-target complexes were amplified in a post-capture PCR reaction. The Illumina NovaSeq 6500 (Illumina, San Diego, CA, USA) was used to sequence the whole transcriptome from patients to an average of 60 M reads. Raw data were demultiplexed by the Illumina Dragen BioIT accelerator (Illumina, San Diego, CA, USA), trimmed, counted, PCR duplicates removed, and aligned to the human reference genome hg19 by the STAR aligner. For transcription counting, transcripts per million values were generated using the Salmon expression pipeline.

### 2.7. Immunotherapy-Related Biomarker Assessment

A combination of multiple test platforms was used to determine the microsatellite instability (MSI) or mismatch repair (MMR) status of the tumors profiled, including fragment analysis (FA, Promega, Madison, WI, USA), IHC (MLH1, M1 antibody; MSH2, G2191129 antibody; MSH6, 44 antibody; and PMS2, EPR3947 antibody [Ventana Medical Systems, Inc., Tucson, AZ, USA]), and NGS (for tumors tested with NextGen Seq or WES, 7000 target microsatellite loci were examined and compared to the reference genome hg19 from the University of California). A tumor was determined MSI-high (MSI-H) by FA if two or more mononucleotides out of the five markers included in the assay were abnormal; a tumor was considered mismatch repair deficient (dMMR) by IHC if complete absence of nuclear protein expression of any of the four proteins was observed; a tumor was considered MSI-H by NGS by a threshold of 46 or more altered loci per tumor. MSI or MMR status of the tumor was determined in the order of IHC, FA, and NGS.

For tumor mutational burden (TMB), a cutoff of 10 mutations/Mb was established, based on the result of the KEYNOTE-158 trial showing clinical activity of pembrolizumab in tumors harboring a TMB ≥ 10 (TMB-H) across a variety of previously treated solid tumors [21].

PD-L1 expression was tested via IHC using SP142 antibody (Spring Biosciences, Pleasanton, CA, USA) and 22C3 (Agilent, New York, NY, USA) with a positive cut-off for >1% staining, according to standard protocol.

### 2.8. Immune Microenvironment

The tumor-infiltrating immune cell landscape was analyzed by quanTIseq (v1.17.0). T cell inflammation scores (TISs) were calculated using a T cell-inflamed gene expression signature. Interferon (IFN) scores were calculated using a validated 18-gene signature including CCL5, CD27, CD274, CD276, CD8A, CMKLR1, CXCL9, CXCR6, HLA-DQA1, HLA-DRB1, HLA-E, IDO1, LAG3, NKG7, PDCD1LG2, PSMB10, STAT1, and TIGIT. MAPK pathway activation scores (MPASs) were calculated using a 10-gene set that was shown to correlate with response to MAPK inhibition and worse clinical outcomes in multiple tumor types (SPRY2, SPRY4, ETV4, ETV5, DUSP4, DUSP6, CCND1, EPHA2, and EPHA4).

### 2.9. Real-World Survival Analysis

Real-world overall survival (OS) information was obtained from insurance claims data. OS was calculated from the time of tissue collection as a surrogate for diagnosis or from the start of treatment with pembrolizumab until the last contact. Patients without contact/claims data for a period of at least 100 days were presumed deceased. Conversely, patients with a documented clinical activity within 100 days prior to the latest data update were censored in the analysis. Kaplan–Meier estimates were calculated for molecularly defined patient cohorts. Hazard ratios (HRs) were determined by the Cox Proportional Hazards model, and *p*-values by the log-rank test. Significance was determined as *p* < 0.05.

### 2.10. Statistical Analysis

The molecular features of VP and VN tumors were compared. Categorical data was assessed using a chi-square or Fisher’s exact test, where appropriate. Continuous data was assessed by a nonparametric Mann–Whitney U test. *p*-values were adjusted for multiple hypothesis testing by Benjamini–Hochberg (*q*-value). All statistical analyses were two-sided at a significance level set to 0.05.

## 3. Results

### 3.1. Patient Characteristics

Viral status was determined for 95 WES-profiled cases: 57 (60%) were VP-MCC and 38 (40%) were VN-MCC (Table 1). Demographics of the patient population included in this study are in Table 1. No significant differences in median age between VP-MCC and VN-MCC patients were noted: VP-MCC 71 (range 22–90) and 75.5 (range 47–90) in the VN-MCC group (*p* = 0.361). VP-MCC and VN-MCC had similar compositions of male (71.9% vs. 76.3%) vs. female (28.1% vs. 23.7%) (*p* = 0.793). No significant differences were noted in the self-reported racial makeup of VP-MCC vs. VN-MCC (Asian American or Pacific Islander: 4.08% vs. 0%; Black or African American: 2.04% vs. 5.88%; White: 85.7% vs. 94.1%; or Other: 8.16% vs. 0%, *p* = 0.162), but VP-MCC patients seemed to be more of Hispanic or Latino ethnicity compared to VN-MCC patients (17.7% vs. 2.94%, *p* = 0.039) (Table 1).

### 3.2. Mutational Differences

**Figure 1:**
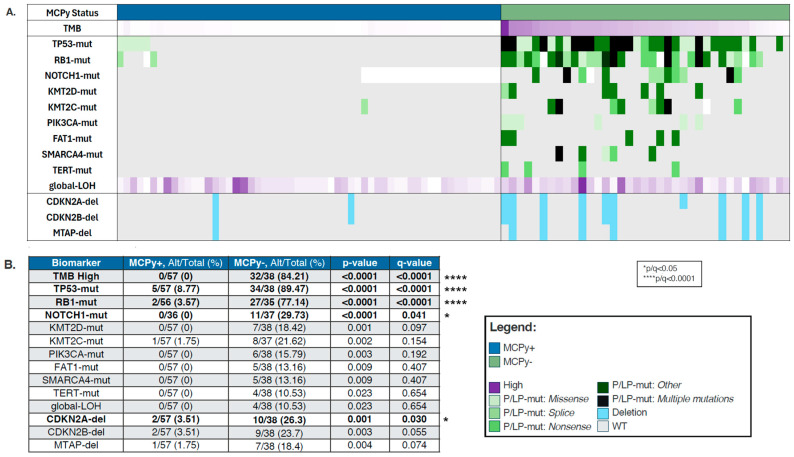
(**A**) Oncoprint depicting molecular differences between VP (blue) and VN (green) Merkel Cell Carcinoma. Purple is located in the legend. Dark purple means high. (**B**) Prevalence by biomarker in VP-MCC and VN-MCC.

VP and VN-MCC samples were analyzed for mutational differences involved in oncogenesis, with findings depicted in an OncoPrint (Figure 1A). VN-MCC was more frequently found to be TMB high (84.21% vs. 0%, *p*-value 0) with median TMB 27.5 Muts/Mb (range 2–98) (Table 1). In line with the high TMB, VN-MCC had a significantly higher prevalence of mutations in *TP53* (89.47% vs. 8.77%, *q* ≤ 0.0001), RB1 (77.14% vs. 3.57%, *q* ≤ 0.0001), and *NOTCH1* (29.73% vs. 0%, *q* = 0.041), with a trend towards higher prevalence of mutations in *KMTD2* (18.42% vs. 0%, *p* = 0.001, *q* = 0.097), *KMT2C* (21.62% vs. 1.75%, *p* = 0.002, *q* = 0.154), PIK3CA (15.79% vs. 0%, *p* = 0.003, *q* = 0.192), *FAT1* (13.16% vs. 0%, *p* = 0.009, *q* = 0.407), *SMARCA4* (13.16% vs. 0%, *p* = 0.009, *q* = 0.407), *TERT* (10.53% vs. 0%, *p* = 0.023, *q* = 0.654), and also in global loss of heterozygosity (gLOH) (10.53% vs. 0%, *p* = 0.023, *q* = 0.654) (Figure 1B). CDKN2A-del were significantly more prevalent in MCPy− tumors compared to MCPy+ tumors (26.3% vs. 3.51%, *q* = 0.030) with similar patterns in CDKN2B-del (23.7% vs. 3.51%, *q* = 0.055) and MTAP-del (18.4% vs. 1.75%). We did not see any significant differences in copy number amplification, PD-L1 positivity by IHC, or dMMR/MSI-H between VP and VN-MCC.

### 3.3. Transcriptomic Immune Signatures

**Figure 2:**
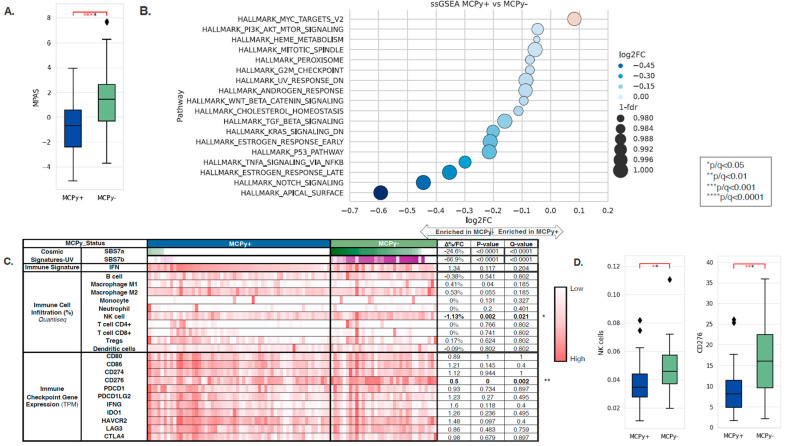
(**A**) MAPK Activity Scores in VP-MCC (blue) and VN-MCC (green) showing greater MPAS in VN disease. (**B**) Single-sample gene set enrichment analysis (ssGSEA) of the Hallmarks of Cancer pathways showed enrichment of MYC targets in VP-MCC compared to VN-MCC. (**C**) COSMIC Mutational Signatures showed a significant difference between VP-MCC and VN-MCC in SBS7a and SBS7b. (**D**) Immune cell deconvolution by quanTIseq of bulk RNA expression showed enrichment of NK cells in VN-MCC compared to VP-MCC.

Transcriptomic and tumor immune microenvironment differences were identified between VP and VN-MCC tumors. MAPK Activity Scores (MPAS) were higher in VN-MCC compared to VP-MCC (−0.66 vs. 1.47, *q* = 0.0001) (Figure 2A). Single-sample gene set enrichment analysis (ssGSEA) of the Hallmarks of Cancer pathways showed enrichment of MYC targets in VP-MCC compared to VN-MCC (NES: 1.06, *q* = 0.003), while VP-MCC had down-regulation of other pathways such as PI3K/AKT/MTOR Signaling, Heme Metabolism, Mitotic Spindle, Peroxisome, G2M Checkpoint, UV Response Down, Androgen Response, WNT/Beta-Catenin Signaling, Cholesterol Homeostasis, TGF-Beta Signaling, KRAS Signaling Down, Estrogen Response Early, P53 Pathway, TNFA Signaling via NFKB, Estrogen Response Late, NOTCH Signaling, and Apical Surface (NES: 0.66–0.97, *q* < 0.05) (Figure 2B). COSMIC Mutational Signatures showed a significant difference between VP-MCC and VN-MCC in two UV signatures: SBS7a (0% vs. 24.6%, *p* ≤ 0.0001) and SBS7b (0% vs. 66.9, *p* ≤ 0.0001), aligning with TMB findings. Immune cell deconvolution by quanTIseq of bulk RNA expression showed enrichment of NK cells in VN-MCC compared to VP-MCC (3.46% vs. 4.59%, *q* = 0.021) (Figure 2C,D). No significant differences were noted in other immune cell fractions nor in the IFN response signature. No significant differences were noted in immune checkpoint gene expression except for *CD276*, where expression was lower in VP-MCC compared to VN-MCC (0.5-FC, *q* = 0.002) (Figure 2C,D).

### 3.4. Clinical Outcomes

**Figure 3:**
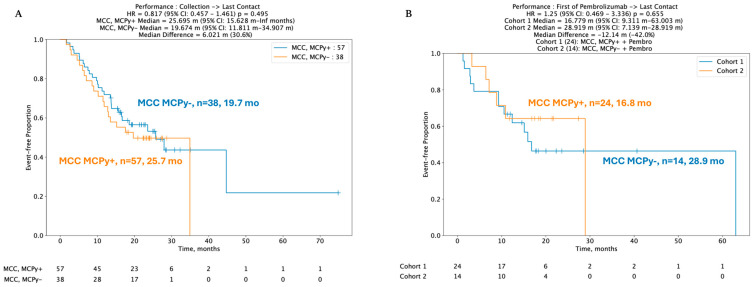
(**A**,**B**) Kaplan–Meir curves depicting no difference in real-world overall survival (rwOS) between VP and VN tumors, including post-pembrolizumab treatment.

Despite key differences in mutation profiles, immune signatures, and gene expression pertinent to immune checkpoints, no statistically significant difference was identified between real-world overall survival (rwOS) and post-pembrolizumab overall survival (OS) between VP and VN-MCC tumors. Median rwOS for 57 patients with VP-MCC tumors was 25.7 months versus 19.7 months in 38 VN-MCC patients (HR 95% CI: 0.817 (0.457–1.46), *p* = 0.495) (Figure 3A). In 38 patients with known viral status and available data regarding ICI treatment with pembrolizumab, median real-world rwOS was 16.8 months in 24 VP-MCC patients and not reached in 14 VN-MCC patients, HR 1.25 (95% CI 0.469–3.336, *p*-value 0.655) (Figure 3B).

## 4. Discussion

We leveraged WES and WTS data to determine mutational differences and differential gene expression in advanced MCC with the aim of identifying features that contribute to ICI resistance and subsequently identifying alternative therapeutic targets. We used WES to corroborate that VN tumors exhibit high TMB, harbor COSMIC UV signatures, and have more mutations in TP53, RB1, and NOTCH1. By leveraging WTS, we determined that MPAS, NK cell infiltration, and the immune checkpoint inhibition gene CD276 are upregulated in VN tumors. Limitations of this study include that the data are retrospective and based on insurance claims. Data was collected from multiple centers, and data pertaining to the staging and sequencing of therapies was not ascertainable. Thus, it is difficult to account for variation in diagnostic criteria between institutions. Data regarding immunosuppression status were also not available. Analysis for some genes, including the high frequency of indeterminate results for NOTCH1 mutations, was incomplete due to dataset limitations. Lastly, real-world overall survival (rwOS) curves depicted in Figure 3A, B are formulated from available follow-up data. These limitations in statistical power likely explain the lack of observed significant difference between post-pembrolizumab VP and VN MCC. Our data provide further evidence that factors contributing to oncogenesis, treatment response, and resistance extend beyond viral status in MCC.

Currently, three ICIs are approved in the United States for frontline treatment of MCC: avelumab, pembrolizumab, and retifanlimab; however, many patients develop resistance or are refractory to therapy. The Phase 2 JAVELIN Merkel 200 study showed an overall response rate (ORR) of 39.7% in 116 treatment-naïve patients with metastatic MCC treated with the PD-L1 inhibitor avelumab. Patients with PD-L1-positive tumors had higher responses with an ORR of 61.9% [7]. Response rates were also higher in patients with VN disease, assessed by IHC. Durable response rate (DRR) was 27.1% in VP tumors and 35.1% in VN tumors. Long-term follow-up showed longer OS in PD-L1-positive tumors, but data regarding viral status were not included. In the Phase 2 KEYNOTE-017 study demonstrating pembrolizumab efficacy in advanced MCC, the ORR was 58% in the total cohort. Among 25 patients for whom response could be evaluated, 62% with VP disease and 44% with VN disease had an objective response [8]. This varies from the recently published findings by Knepper et al., who found that VP and VN-MCC had a 41% (9/22) and 50% (7/14) response rate to ICI, respectively. These authors also found that PD-1, but not PD-L1 expression, correlated with ICI response [22]. The PODIUM-201 trial demonstrated that retifanlimab, a PD-1 monoclonal antibody, also demonstrated clinical activity in advanced MCC; however, data regarding efficacy in VP and VN subgroups are not yet published, and the drug is not as commonly used in clinical practice [23]. While we exercise caution in cross-trial comparison, and the sample size was significantly smaller in KEYNOTE-017, these three practice-changing clinical trials did not definitively provide insight into viral status and ICI response. Our data did not demonstrate a statistically significant difference between ICI responsiveness by viral status; however, as discussed above, limitations of our rwOS data include limited follow-up.

Additional studies have aimed to overcome ICI resistance [9,10,24]. The combination of the anti-cytotoxic T-lymphocyte antigen 4 (CTLA-4) monoclonal antibody ipilimumab in combination with nivolumab in avelumab-refractory disease has been studied [25,26]. The TRICK-MCC study aimed to combine anti-PD-1, anti-lymphocyte-activating gene 3 (LAG-3), and anti-T-cell immunoglobulin and mucin domain-containing protein 3 (TIM-3) drugs to overcome anti-PD-1 resistance but was stopped early with 12 patients enrolled [27]. The ongoing MATRiX trial combines the ataxia telangiectasia and Rad3-related (ATR) inhibitor tuvusertib with avelumab in ICI-refractory MCC [28]. While mechanisms and approaches for overcoming checkpoint resistance are actively under study, there is also a need for larger scales to identify post-ICI treatments [29,30]. Here, we aim to identify additional molecular targets in MCC through WES, which has not previously been employed in MCC.

Through transcriptomic analysis of NK cells and MPAS, our findings implicate alternate therapeutic targets. NK cells are an attractive option for cancer immunotherapy due to their intrinsic targeting of stressed cells and are in various stages of development for other solid tumor and hematologic malignancies [31]. Increased NK cell infiltration in VN-MCC may represent a new pathway for therapeutic immune modulation. Studies such as the QUILT 3.009 and QUILT 3.063 trials were underway to assess the efficacy of activated NK cells and IL-15 agonists in MCC that is at an advanced stage or has progressed after ICI, but both studies have since been terminated due to low recruitment [32,33]. CD276 (also known as B7-H3), shown here to be upregulated in VN-MCC compared to VP-MCC, is under ongoing investigation in other solid tumor malignancies, including prostate and breast cancer [34,35,36]. In vitro studies and RNA sequencing have identified aberrations in the MAPK pathway, which encompasses the RAS-RAF-MEK signaling cascade, in MCC cell lines [37,38,39]. While this implicates potential therapeutic targets, studies are limited to the preclinical setting, small sample size, and extraction techniques [15]. Further investigation is needed to definitively characterize the role of RAF and MEK inhibitor efficacy in MCC.

## 5. Conclusions

While much clinical progress has been achieved in advanced and metastatic MCC with ICI treatment, MCC remains an aggressive malignancy with poor outcomes. There remains a need to identify alternate targets in cases of resistant, refractory, and recurrent disease. Using WES and WTS, we demonstrated an increase in mutations in *TP53*, *RB1*, *NOTCH1*, *KMTD2*, and *KMT2C*, and an upregulation of MAPK Pathway Activity Score, NK cell infiltration, and the immune checkpoint gene *CD276* in VN-MCC tumors. Our findings underscore the promise of transcriptomic analysis to identify new therapeutic targets for ICI-refractory MCC.

## Figures and Tables

**Table 1:** Patient Demographics.

Characteristic	MCPyV+	MCPyV−	*p*-Value
*n* (%)	57 (60)	38 (40)	
Age, median (range)	71 (22–90)	75.5 (47–90)	0.361
TMB, median (range)	1 (0–7)	27.5 (2–98)	<0.0001
Sex, *n* (%)			
Male	41 (71.9)	29 (76.3)	0.793
Female	16 (28.1)	9 (23.7)	
Biopsy Site, *n* (%)			
Skin	17 (28.1)	19 (50)	0.109
Lymph Nodes	17 (29.8)	10 (26.3)	
Other Mets	23 (40.4)	9 (23.7)	
Race, *n* (%)			
Asian American or Pacific Islander	2 (4.08)	0 (0)	0.162
Black or African American	1 (2.04)	2 (5.88)	
White	42 (85.7)	32 (94.1)	
Other	4 (8.16)	0 (0)	
Ethnicity, *n* (%)			
Hispanic or Latino	9 (17.7)	1 (2.94)	0.039
Not Hispanic or Latino	42 (82.4)	33 (97.1)	

## Data Availability

The raw data supporting the conclusions of this article will be made available by the authors on request.

## Abbreviations

The following abbreviations are used in this manuscript:

dMMR	Mismatch Repair Deficient
ICI	Immune Checkpoint Inhibitor
MCC	Merkel Cell Carcinoma
MCPyV	Merkel Cell Polyomavirus
MMR	Mismatch Repair
MSI-H	Microsatellite Instability High
MSS	Microsatellite Stable
NGS	Next-Generation Sequencing
NK	Natural Killer
ORR	Objective Response Rate
PD-L1	Programmed Cell Death Ligand 1 (PD-L1)
rwOS	Real-World Overall Survival
TIS	T-Cell Inflammation Scores
TMB	Tumor Mutational Burden
UVR	Ultraviolet Radiation
VN-MCC	Merkel Cell Polyomavirus-Negative Merkel Cell Carcinoma
VP-MCC	Merkel Cell Polyomavirus-Positive Merkel Cell Carcinoma
WES	Whole Exome Sequencing
WTS	Whole Transcriptome Sequencing
